# Factors affecting adherence to evidence-based guidelines in the treatment of URI, sinusitis, and pharyngitis

**DOI:** 10.3402/jchimp.v3i2.20744

**Published:** 2013-07-05

**Authors:** Andrew Crocker, Richard Alweis, Jorge Scheirer, Shannon Schamel, Tom Wasser, Kris Levingood

**Affiliations:** 1 The Reading Hospital and Medical Center, West Reading, PA; 2 Jefferson Medical College, Thomas Jefferson University, Philadelphia, PA; 3 Consult-Stat: Complete Statistical Services, Macungie, PA; 4 Rochester Institute of Technology, Rochester, NY

**Keywords:** guideline adherence, outpatient antibiotic prescribing, outpatient practice quality

## Abstract

**Introduction:**

Sinus infections, sore throats, and upper respiratory tract infections (URI) are common reasons patients seek medical care. Well-established treatment guidelines exist for prescribing antibiotics in these clinical scenarios, but are not often followed.

**Objective:**

The objective of this study is to determine practice patterns related to prescribing antibiotics for sinusitis, URI, and pharyngitis. The main hypothesis is that attending physicians improve their adherence to antibiotic guidelines with a learner present.

**Methods:**

A retrospective cohort study was performed on patients treated for URI, sinusitis, and pharyngitis at an ambulatory faculty practice. The use of relevant ICD-9 codes from January 1, 2008 to January 30, 2012 resulted in 1,548 patient encounters which were reviewed for guideline adherence. Univariate analysis and multivariate logistic regression was performed for each outcome variable to determine if they influence antibiotic adherence. Variables studied were physician, presence of a learner, BMI, age, gender, day of the week, month, diabetes, immunosuppression, and COPD.

**Results:**

Multivariate analysis showed the statistically significant variables were age (p=0.038) for pharyngitis and provider (p=0.013) for URI. There were no significant findings for sinusitis. Guideline adherence was 24% in patients with pharyngitis, 42% in acute sinusitis, 79% in URI, and 57% overall.

**Conclusion:**

Guideline adherence varies depending on the treating physician and decreases when treating younger patients with pharyngitis. The presence of a learner did not improve prescribing habits. The reason for these findings remain unclear, but considerations for improvement could include following antibiotic adherence as a quality measure, giving patients handouts educating them about the impact of overprescribing antibiotics, and further education amongst faculty and residents about adhering to nationally recognized guidelines.

Complaints of sinus infections, sore throats, and upper respiratory tract infection (URI) are common. Sore throats alone account for 1–2% of all hospital visits in the United States (1–3). Well-established treatment guidelines (Table 1) exist for the usage of antibiotics in these clinical scenarios, but they are often not followed (4–7). For example, a national survey showed that 73% of adults with sore throat received antibiotics between 1989 and 1999 in the United States (8), far higher than would be expected with the established guidelines. Further, the study also revealed that antibiotics not recommended as first line treatment for streptococcal pharyngitis were prescribed in 68% of the inappropriately treated patients (8).

**Table 1:** Antibiotic prescribing guidelines

Diagnosis	Guideline
Pharyngitis	Bisno et al. Clin Infect Dis 2002
Upper respiratory tract infection	Wong et al. Am Fam Physician 2006
Sinusitis	Chow et al. Otolaryngol Head Neck Surg 2007

It is reasonable to assume that clinician–educators, in the presence of a learner, are more likely to adhere to treatment guidelines than non-educators. However, the data on the ‘July phenomenon’ and the general question comparing the quality of care in teaching versus non-teaching hospitals are mixed. Overall, several recent reviews concluded that major teaching hospitals had better quality outcomes than minor teaching hospitals, who themselves had better quality outcomes than non-teaching hospitals (9, 10). However, the authors of these reviews noted that the outcomes measured in the reviewed studies were not at the target levels at any of the hospitals in the studies (9, 10). None of the studies addressed whether the presence of the learner full-time in the hospital was the reason for the improved outcomes or whether the presence of the learner had a positive influence on the teaching faculty for guideline adherence.

This study evaluated the antibiotic prescribing behaviors of an internal medicine faculty at a university-affiliated community residency program in the management of patients with URI, acute sinusitis, and acute pharyngitis and whether the presence of a learner affected guideline adherence.

## Methods

### Data acquisition

**Fig. 1:**
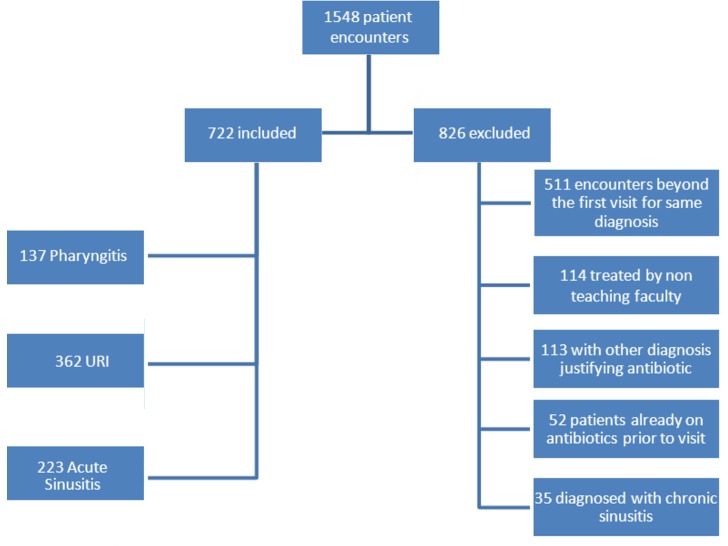
Patient population.

A retrospective cohort study was performed on diagnosed cases of URI, sinusitis, and pharyngitis in the aforementioned internal medicine faculty practice. Patients were identified using Centricty (GE Healthcare, London, England) electronic medical records by searching ICD-9 codes for diagnoses of URI (ICD-9 code 465.9), sinusitis (ICD-9 code 465.9), sinusitis (ICD-9 codes 461.8, 461.9, 473.9), and sore throat/pharyngitis (ICD-9 codes 034.0, 462.0). A total of 1,548 patient encounters were identified from January 1, 2008, to January 30, 2012, using one of these codes. The study included 722 patients aged over 18 who had a diagnosis code under investigation. Exclusion criteria were met for 826 patient encounters (Fig. 1). A trained research associate and the physician authors performed the chart review to determine whether the chosen guidelines were appropriately followed. The research associate and a physician author reviewed the first 10% of all cases simultaneously, as well as every 10th case thereafter to ensure agreement between observers.

### Statistical analysis

In order to determine if the combination of demographic and clinical variables were predictive of adherence guidelines, a combination of univariate and multivariate analysis were performed. Univariate analysis was intended to identify those variables that were associated for inclusion in a multivariate logistic regression with ‘guidelines met’ as the dichotomous outcome variable. In order to be included in the analysis, individual providers needed to be included on no fewer than 30 individual patient cases.

Univariate analysis was completed using a combination of clinical, treatment, and demographic variables, and was completed separately for each guideline (sinusitis, pharyngitis, and URI), while the variables for each model were the same. Discrete variables were gender, provider, day of the week, month of the year, diagnosis of chronic obstructive pulmonary disease (COPD), diabetes, immunosuppression, and presence of a learner. Chi-square analysis was performed on these discrete variables. Continuous variables included age and patient body mass index (BMI) and were analyzed with group *t*-test. Any variable with a *p*-value of less than 0.05 was included in the logistic regression.

Multivariate logistic regression was then completed using the ‘Enter’ method and the results included the initial model prediction, end of model prediction, as well as the *p*-value. The model R^2^ is also reported for the final model.

## Results

Table 2 presents the results of the univariate analysis for sinusitis. There were no variables that met the inclusion criteria for the logistic regression. Month of the year was the most closely associated parameter whether or not the guidelines were followed (*p*=0.092); however, no other variables were trend significant for the sinusitis variable. Based on these results, no logistic regression was performed.

**Table 2:** Univariate analysis for sinusitis guidelines (continuous and discrete variables)

	Guidelines met			
			
Variable	No Mean±SD	Yes Mean±SD		*p*
Age	49.3±14.7	52.0±15.3		0.186
Body mass index	30.2±6.9	29.6±7.2		0.601
		No	Yes	
Variable	Category	Count (%)	Count (%)	
Gender	Female	90 (57.0)	68 (43.0)	0.529
	Male	40 (61.5)	25 (38.5)	
Provider	Attending 1	37 (57.8)	27 (42.2)	0.833
	Attending 2	73 (59.8)	49 (40.2)	
	Attending 3	16 (51.6)	15 (48.4)	
	Attending 4	4 (66.7)	2 (33.3)	
Day of week	Monday	14 (63.6)	8 (36.4)	0.739
	Tuesday	35 (54.7)	29 (45.3)	
	Wednesday	17 (56.7)	13 (43.3)	
	Thursday	44 (63.8)	25 (36.2)	
	Friday	20 (56.7)	18 (43.3)	
Month	January	11 (47.8)	12 (52.2)	0.092
	February	14 (70.0)	6 (30.0)	
	March	20 (62.5)	12 (37.5)	
	April	4 (28.6)	10 (71.4)	
	May	13 (56.5)	10 (43.5)	
	June	7 (46.7)	8 (53.3)	
	July	9 (75.0)	3 (25.0)	
	August	4 (50.0)	4 (50.0)	
	September	11 (61.1)	7 (38.9)	
	October	8 (72.7)	3 (27.3)	
	November	18 (78.3)	5 (21.7)	
	December	11 (44.0)	14 (56.0)	
COPD	No	124 (58.8)	87 (41.2)	0.549
	Yes	6 (50.0)	6 (50.0)	
Diabetes	No	113 (57.7)	83 (42.3)	0.600
	Yes	17 (63.0)	10 (37.0)	
Immunosuppression	No	122 (57.3)	91 (42.7)	0.154
	Yes	8 (80.0)	2 (20.0)	
Learner	No	112 (58.9)	78 (41.1)	0.636
	Yes	18 (54.5)	15 (45.5)	

Table 3 presents the results for the univariate analysis for pharyngitis. Several variables met the inclusion criteria for the logistic regression: provider (*p*<0.001), diabetes (*p*=0.030), immunosuppression (*p*=0.033), learner (*p*=0.048), and patient age (*p*<0.001). All other variables (gender, day of the week, COPD, and patient BMI) were not significant and not included in the logistic regression.

**Table 3:** Univariate analysis for pharyngitis guidelines (continuous and discrete variables)

	Guidelines met			
			
Variable	No Mean±SD	Yes Mean±SD		*p*
Age	41.4±15.9	54.0±19.7		<0.001
Body mass index	28.6±7.2	29.3±4.6		0.637
		No	Yes	
Variable	Category	Count (%)	Count (%)	
Gender	Female	71 (77.2)	21 (22.8)	0.622
	Male	33 (73.3)	12 (26.7)	
Provider	Attending 1	10 (45.5)	12 (54.5)	<0.001
	Attending 2	36 (80.0)	9 (20.0)	
	Attending 3	54 (87.1)	8 (12.9)	
	Attending 4	4 (50.0)	4 (50.0)	
Day of week	Monday	26 (86.7)	4 (13.3)	0.154
	Tuesday	25 (86.2)	4 (13.8)	
	Wednesday	16 (72.7)	6 (27.3)	
	Thursday	22 (66.7)	11 (33.3)	
	Friday	15 (65.2)	8 (34.8)	
Month	January	15 (83.3)	3 (16.7)	0.478
	February	12 (75.0)	4 (25.0)	
	March	9 (81.8)	2 (18.2)	
	April	8 (80.0)	2 (20.0)	
	May	10 (71.4)	4 (28.6)	
	June	7 (70.0)	3 (30.0)	
	July	6 (75.0)	2 (25.0)	
	August	6 (46.2)	7 (53.8)	
	September	9 (90.0)	1 (10.0)	
	October	7 (87.5)	1 (12.5)	
	November	3 (60.0)	2 (40.0)	
	December	12 (85.7)	2 (14.3)	
COPD	No	97 (75.8)	31 (24.2)	0.892
	Yes	7 (77.8)	2 (22.2)	
Diabetes	No	94 (79.0)	25 (21.0)	0.030
	Yes	10 (55.6)	8 (44.0)	
Immunosuppression	No	91 (73.4)	33 (26.6)	0.033
	Yes	13 (100.0)	0 (0.0)	
Learner	No	93 (78.8)	25 (21.2)	0.048
	Yes	11 (57.9)	8 (24.1)	

Table 4 presents the results for URI. Only two variables were statistically significant, provider (*p*<0.001) and patient age (*p*=0.020). Day of the week was trend significant (*p*=0.053) but was not included in the logistic regression.

**Table 4:** Univariate analysis for URI guidelines (continuous and discrete variables)

	Guidelines met			
			
Variable	No Mean±SD	Yes Mean±SD		*p*
Age	58.3±15.9	53.1±17.5		0.020
Body mass index	30.3±7.9	29.6±7.4		0.479
		No	Yes	
Variable	Category	Count (%)	Count (%)	
Gender	Female	49 (22.5)	169 (77.5)	0.310
	Male	26 (18.1)	118 (81.9)	
Provider	Attending 1	38 (50.0)	38 (50.0)	<0.001
	Attending 2	6 (9.1)	60 (90.9)	
	Attending 3	30 (18.6)	131 (81.4)	
	Attending 4	1 (1.7)	58 (98.3)	
Day of week	Monday	15 (19.2)	63 (80.8)	0.053
	Tuesday	15 (20.0)	60 (80.0)	
	Wednesday	17 (21.0)	64 (79.0)	
	Thursday	6 (10.2)	53 (89.8)	
	Friday	22 (31.9)	47 (68.1)	
Month	January	7 (15.6)	38 (84.4)	0.144
	February	4 (13.8)	25 (86.2)	
	March	12 (24.5)	37 (75.5)	
	April	7 (21.2)	26 (78.8)	
	May	3 (11.5)	23 (88.5)	
	June	11 (39.3)	17 (60.7)	
	July	5 (33.3)	10 (66.7)	
	August	1 (10.0)	9 (90.0)	
	September	3 (23.1)	10 (76.9)	
	October	5 (13.2)	33 (86.8)	
	November	4 (11.8)	30 (88.2)	
	December	13 (31.0)	29 (69.0)	
COPD	No	63 (19.7)	257 (80.3)	0.182
	Yes	12 (28.6)	30 (71.4)	
Diabetes	No	62 (20.7)	237 (79.3)	0.986
	Yes	13 (20.6)	50 (79.4)	
Immunosuppression	No	71 (20.3)	278 (79.7)	0.362
	Yes	4 (30.8)	9 (62.2)	
Learner	No	64 (21.3)	237 (78.7)	0.570
	Yes	11 (18.0)	50 (82.0)	

### Multivariate analysis

For pharyngitis, the only significant variable was age (*p*=0.038). The initial model prediction was 77.7% accuracy with all patients being pushed into the ‘guidelines not being met’ category. Final model prediction was 87.4% with the model correctly identifying 97 of 104 patients (93.3%) that did not meet guideline criteria and only 14 of 31 patients (45.2%) that were correctly predicted to meet guidelines given the inclusion variables. The overall model R2 was 0.484 indicating 48.4% of all the variance can be explained by the combination of these variables.

For URI, only two variables were entered, and both were statistically significant. Provider (*p*=0.013) and a trend significance for gender (*p*=0.054). There was minimal improvement in predictability from 78.0 to 80.6% after model calculation. There were 320 patients included in this model. Final model R2 was 0.296, or 29.6%, of all the variance being explained by the combination of these two variables.

## Discussion

This retrospective cohort study shows a disappointing overall weighted average of 57% adherence to guidelines for pharyngitis, URI, and acute sinusitis. Guideline adherence for each diagnosis was 24% in patients with pharyngitis, 42% in acute sinusitis, and 79% in URI. In pharyngitis alone, univariate analysis indicated that the presence of a learner positively affected attending behavior, whereas diabetes and immunosuppression negatively affected attending behavior; however, none of these variables were supported in the multivariate analysis. Within the practice, in the univariate analysis, the widest variability in attending guideline adherence was seen in URI, which was confirmed in the multivariate analysis. In addition, the logistic regression does show that younger age of patient is associated with lack of guideline adherence for treatment of pharyngitis. Patient comorbidities with COPD, diabetes, or immunosuppression did not significantly alter the provider prescribing habits.

The low rate of adherence with antibiotic prescribing guidelines was likely influenced by several factors. Providers may not be familiar with or may not agree with the guidelines. Furthermore, the time-pressured environment of primary care does not lend itself to the application of complex multi-step guidelines, which might lead to over-estimation of risk of bacterial infection or benefits of antibiotic therapy. In addition, it often takes longer to explain to patients that antibiotics are not necessary than it does to actually generate a prescription. The current focus on patient satisfaction may increase the pressure on providers to meet perceived patient expectations for antibiotic therapy for their symptoms. Finally, it is conceivable that provider desire to reduce the perceived risk of callbacks if an antibiotic was not prescribed was a factor in their decision.

This study had several limitations. The knowledge and acceptance of treatment guidelines by practitioners at the time of treatment could not be measured in this retrospective study. One of the providers included in the study left the practice shortly after the conclusion of the study, so querying providers regarding baseline knowledge would not have been feasible. In addition, there were 36 patients on chronic immunosuppressive therapy, all of whom received antibiotics for their infections. We did not exclude these patients because the guidelines do not specifically make recommendations regarding care of these patients. Nonetheless, many providers empirically prescribe antibiotics for immunosuppressed patients with respiratory and pharyngeal infections. Finally, as this was a study in a single primary care practice, there is limited generalizability to other venues and larger organizations.

Considerations to improve guideline adherence in the future could include following adherence as a quality measure, patient handouts educating them on the impact of inappropriate antibiotics (11), and further education among the faculty and residents regarding current guidelines. Residency venues that may be appropriate include a morning report or a noon conference.

## Conclusion

This retrospective cohort study showed a disappointing 57% overall adherence to guidelines for pharyngitis, URI, and acute sinusitis. Surprisingly, the presence of a learner did not positively affect the guideline adherent behavior of attending physicians. The reasons for this remain unclear. Given the complications of unnecessary antibiotic therapy are significant, further study is needed to determine the best modality to assist physicians in adhering to nationally recognized guidelines.
